# Dietary Differences in Male Workers among Smaller Occupational Groups within Large Occupational Categories: Findings from the Japan Environment and Children’s Study (JECS)

**DOI:** 10.3390/ijerph15050961

**Published:** 2018-05-11

**Authors:** Rie Tanaka, Mayumi Tsuji, Ayako Senju, Koichi Kusuhara, Toshihiro Kawamoto, Japan Environment and Children’s Study Group

**Affiliations:** 1 Department of Environmental Health, School of Medicine, University of Occupational and Environmental Health, 1-1 Iseigaoka, Yahatanishi-ku, Kitakyushu, Fukuoka 807-8555, Japan; t-rie@med.uoeh-u.ac.jp (R.T.); kawamott@med.uoeh-u.ac.jp (T.K.); 2 Japan Environment and Children’s Study, UOEH Subunit Center, University of Occupational and Environmental Health, 1-1 Iseigaoka, Yahatanishi-ku, Kitakyushu, Fukuoka 807-8555, Japan; senju-a@med.uoeh-u.ac.jp (A.S.); kkusuhar@med.uoeh-u.ac.jp (K.K.); 3 Department of Pediatrics, School of Medicine, University of Occupational and Environmental Health, 1-1 Iseigaoka, Yahatanishi-ku, Kitakyushu, Fukuoka 807-8555, Japan

**Keywords:** occupational classification, dietary intake, nutrient intake

## Abstract

Studies examining workers’ diet according to smaller occupational groups within “large occupational categories” are sparse. The aim of this study was to examine the potential differences in workers’ diets based on the classification of workers into smaller occupational groups that comprise “large occupational categories”. The subjects of this study were working fathers who had participated in the Japan Environment and Children’s Study (N = 38,656). Energy and nutrient intake were calculated based on data collected from the Food Frequency Questionnaire. Occupations were classified according to the Japanese Standard Occupational Classification. Logistic regression analyses were performed to examine the adherence to current dietary recommendations within smaller occupational groups. In particular, significant differences were observed among the categorical groups of “professional and engineering workers”, “service workers”, and “agricultural, forestry, and fishery workers”. In “professional and engineering workers”, teachers showed higher odds of adherence to calcium intake recommendations compared with nurses (OR, 2.54; 95% CI, 2.02–3.14; *p* < 0.001). In “agricultural, forestry, and fishery workers”, agriculture workers showed higher odds of adherence to calcium (OR, 2.15; 95% CI, 1.46–3.15; *p* < 0.001) and vitamin C (OR 1.90, 95% CI 1.31–2.74, *p* = 0.001) intake recommendations compared with forestry and fishery workers. These findings may be beneficial from a research perspective as well as in the development of more effective techniques to improve workers’ diet and health.

## 1. Introduction

Dietary considerations to prevent various health problems, such as cardiovascular diseases [1,2,3,4,5], diabetes, and obesity [5], have been widely discussed. To prevent or improve such lifestyle-related problems, occupational health staff have been involved in giving advice about food and nutrition intake to workers. There are various interventions that have been implemented in the workplace, including individual nutrition education and behavior change approaches related to food choice [6]. The promotion of a healthy diet in workplaces could be particularly important for men, as they are reported to have less nutritional knowledge than women [7]. In Japan, the labor force participation rate for 15–64 year-olds was 76.9% in 2016 [8]. Moreover, men are more likely to work longer hours than women [9]. Therefore, the workplace is a suitable and appropriate environment to promote the benefits of a healthy diet, especially among men.

Dietary behavior and intake have been found to vary across different occupations [10,11,12,13,14,15]. Most studies have classified the occupational status of participants using “broad occupational categories” such as “professionals” [16], “managerial staff” [10], “service worker” [12], and “manual worker” [10,13]. For example, a previous study in Japan reported that male workers engaged in “service work”, “transport”, and “labor” were more likely to have poor dietary habits, including no regular meals, no balanced meals, no bland (less salty) meals, and overeating compared with those in “professional work” [14]. Another study in France reported that male “manual workers” consume more cream desserts than “managerial staff” [15]. Similarly, a study in Norway suggested that “professionals, administrators, and officials” were less likely to consume what was termed as a “Western” food pattern, such as French fried potatoes, hot dogs, hamburgers, and so on, and were more likely to consume a “prudent” pattern, such as fruit, vegetables, dishes with fish, and so on, than “manual workers” [13].

However, in such broad occupational categories, there may still be some differences in dietary intake among workers when categorized into smaller and more specific occupational groups. One good example is the category of “professionals”, which includes various kinds of occupations, such as nurses, doctors, and teachers. “Professional workers” generally appear to show healthier dietary characteristics, although health professionals, such as doctors and nurses (which fall under the “professional workers” category), tended to show more risky health behaviors compared with “office workers” [17]. Considering these findings, it is questionable whether all of the professional workers show the same trend in dietary intake. More research is needed to classify workers not only according to broad, large occupational categories, but also according to smaller occupational groups. As of now, the dietary characteristics of the smaller occupational groups remain unknown.

The aim of this study was to investigate the dietary differences among smaller occupational groups within larger occupational categories as typically defined in previous research. This is the first study examining male workers’ diets in a large population according to smaller, more specific occupational group categories, as assessed with the Japan Standard Occupational Classification (Rev. 5, December 2009) [18,19]. Data were derived from the Japan Environment and Children’s Study (JECS).

## 2. Materials and Methods

### 2.1. Study Design

This cross-sectional study used baseline data from the JECS, a prospective observational cohort study designed to examine the effects of environmental factors on children’s health. More than 100,000 pregnant women were recruited from January 2011 to March 2014, with the choice of optional participation for their partner. The detailed study protocol have been described previously [20]. The current study is based on the dataset of jecs-ag-ai-20160424, which was released in June 2016 [21,22].

### 2.2. Ethical Statement

The JECS was approved by the Institutional Review Board of the Japan National Institute for Environmental Studies (Approval number: 2017-002), and the ethics committees of all participating institutions. The study was conducted according to the principles of the Declaration of Helsinki and other national regulations. Each participant gave written informed consent [20].

### 2.3. Study Sample

**Figure 1:**
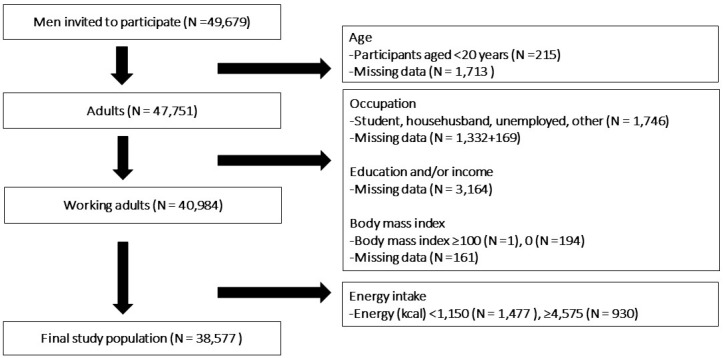
Participant inclusion flowchart.

All of the data, except for household income and educational level, were obtained using self-administered questionnaires completed by fathers (partners) during the first trimester and second/third trimester of their partner’s pregnancy. Information about household income and educational level were obtained from self-administered questionnaires completed by women during the second or third trimester. Exclusion criteria were as follows: under 20 years, students, househusbands, unemployed, or workers not otherwise classifiable. Fathers with missing questionnaire data were also excluded from analyses. In addition, fathers who reported consumption of fewer than 1150 kcal per day or equal to or more than 4575 kcal per day (less than half the energy requirement for the lowest physical activity category or equal to or more than 1.5 times the energy requirement for the highest physical activity category among men aged 18–49 years, respectively, in accordance with the Dietary Reference Intakes for Japanese, 2015 [23,24]) were also excluded. A total of 38,656 males were included in the analysis. The selection of the final study population is described in Figure 1.

### 2.4. Dietary Intake

Participants completed the Food Frequency Questionnaire, which was also used in the Japan Public Health Center-Based Prospective Study for the Next Generation [25]. Fathers were asked how often they had consumed certain types of food and drink, on average, in the previous year, with nine possible responses ranging from “never or less than once per month” to “at least seven times per day”. Energy and nutrient intake were calculated based on the food consumption data. Nutrient intake was evaluated using estimated average requirements (EAR) and tentative dietary goals for preventing lifestyle-related diseases (DG) from the Dietary Reference Intakes for Japanese (2015) [24]. The DG were used as recommendations for protein, fatty acid, carbohydrate, dietary fiber, saturated fatty acid, salt, and potassium. The lowest value for EAR in men aged 18–69 years was used as recommendation for vitamin A, vitamin B_1_, vitamin B_2_, niacin, vitamin B_6_, vitamin B_12_, folic acid, vitamin C, calcium, magnesium, iron, zinc, copper, iodine, selenium, and molybdenum. The estimated energy requirement (EER) was fixed at 2650 kcal (specifically, EER for men aged 18–49 years with moderate physical activity levels). Energy intake from protein, fatty acid, and carbohydrates was calculated as follows: protein (% energy) = protein (g) × 4 (kcal/g)/total energy (kcal) × 100; fatty acid (% energy) = fatty acid (g) × 9 (kcal/g)/total energy (kcal) × 100; carbohydrates (% energy) = (g) × 4 (kcal/g)/total energy (kcal) × 100. Other nutrient intakes were evaluated using energy-adjusted nutrient intake values: energy-adjusted nutrient intake (amount/day) = (reported nutrient intake (amount/day) × estimated energy requirement (EER) (kcal/day)/observed energy intake (kcal/day)).

### 2.5. Socioeconomic Factors

Educational level was categorized into seven groups: junior high school, high school, higher professional school, professional school, junior college, university, and graduate school. Annual income was categorized into nine groups (in yen): <2 million, 2.0–3.9 million, 4.0–5.9 million, 6.0–7.9 million, 8.0–9.9 million, 10.0–11.9 million, 12.0–14.9 million, 15.0–19.9 million, and ≥20 million. Occupation was classified using the Japanese Occupational Classification (Rev. 5, December 2009) [18], which contains 12 Major Groups of workers: administrative and managerial; professional and engineering; clerical; sales; service; security; agricultural, forestry, and fishery; manufacturing; transport and machine operation; construction and mining; carrying, cleaning, packaging, and related work; and workers not classifiable by occupation (who were not included in the present analysis). Importantly, occupation was classified into smaller and more specific groups using the more detailed classification based on the Minor Groups and Unit Groups of the Japanese Standard Occupational Classification (Rev. 5, December 2009) [18,19]. Very small groups—namely, those composed of less than 1.0% of all participants—were integrated into other groups, or unified within each Major Group. For example, within the group of “administrative and managerial workers”, smaller groups consisting of less than 1.0% of all participants were integrated into a category for other administrative and managerial workers. Within “professional and engineering workers”, smaller groups consisting of less than 1.0% of all participants were integrated into a category for other specialist professionals. Within “transport and machine operation workers”, smaller groups consisting of less than 1.0% of all participants, including stationary and construction machinery operators, were integrated into a category for other transport workers. Forestry and fishery workers were unified. Carrying, cleaning, and packaging workers were also unified. On completion, all of the participants had been classified into a total of 39 occupational groups.

### 2.6. Statistical Analysis

Data regarding energy and some of the nutrient intake (calcium and vitamins A and C) were transformed using natural logarithms because the distribution of continuous variables was skewed. Analysis of variance (ANOVA) and *t*-tests were carried out to examine the differences among the occupations that had been classified into the same Major Groups. Logistic regression analysis was performed to examine the association between adherence to dietary recommendations and smaller occupational group assignment within each Major Group, using the occupation that showed the lowest intake as the reference category, which was then adjusted for age, body mass index, household income, and educational level. Energy and nutrient intakes, age, and body mass index were analyzed as continuous variables, while occupation, household income, and educational level were analyzed as categorical variables. Levels of significance were represented by *p* values. Data were analyzed using Stata/IC 14.0 (StataCorp., College Station, TX, USA).

## 3. Results

Table 1 and Table 2 show the characteristics of the study participants. The mean age was 33.0 (±5.7) years (data not shown). The most common occupations were “professional and engineering workers” (*n* = 12,307; 31.8%). Approximately one-third of the participants had graduated from a university. More than half reported having an annual household income as equal to, or more, than four million yen. Approximately three-fifths of the participants met the recommendation for energy intake from carbohydrate, while only 6% participants met the recommendations for dietary fiber intake. Regarding some micronutrients (i.e., vitamin B_2_, niacin, vitamin B_6_, vitamin B_12_, folic acid, magnesium, iron, zinc, copper, iodine, selenium, and molybdenum), the median values among all of the smaller occupational groups met the recommendations; however, this study did not include a detailed analysis.

**Figure 2:**
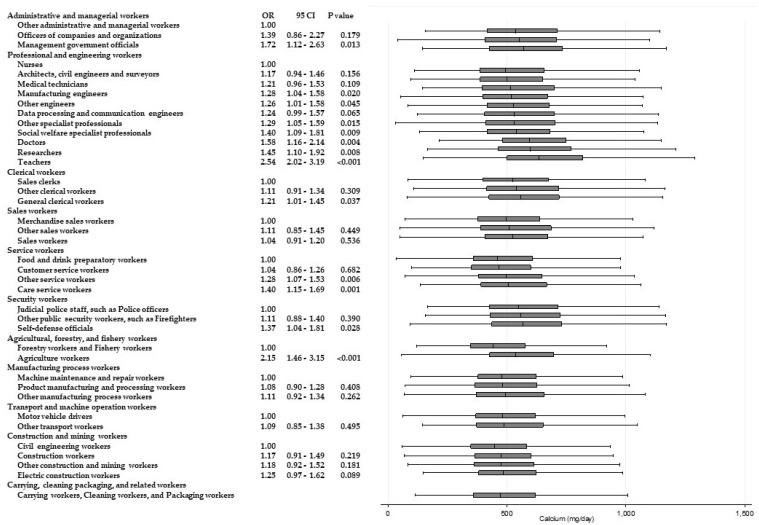
Daily calcium intake according to occupational groups. OR: Odds ratio of daily calcium intake equal to or more than 550 mg/day.

**Figure 3:**
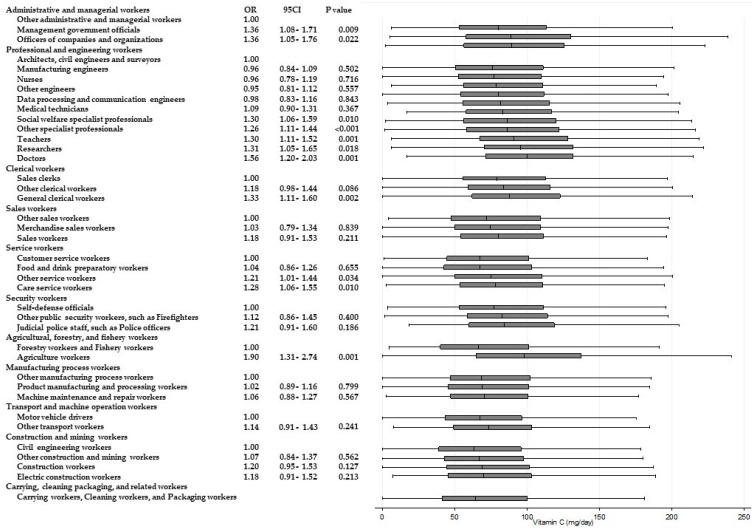
Daily vitamin C intake according to occupational groups. OR: Odds ratio of daily vitamin C intake equal to or more than 85 mg/day.

Table 3 shows the results of ANOVAs and *t*-tests examining the differences in energy and nutrient intake between occupations within the same Major Groups. Significant differences (*p* < 0.001) for all of the items were observed among “professional and engineering workers”. Similarly, significant differences in eight items were observed among “service workers”, and significant differences in six items were observed among “agricultural, forestry, and fishery workers”, respectively. Across these Major Groups, there were significant differences among participants’ intake of dietary fiber and micronutrients such as potassium, calcium, and vitamins A, B_1_, and C. For calcium and vitamin C intake, significantly higher odds of adherence to recommendations were found. Figure 2 and Figure 3 present box and whisker plots demonstrating the associations between occupations and calcium and vitamin C intake, respectively. The figures also show the findings from logistic regression analysis for adherence to dietary recommendations, using the lowest intake group within each Major Group as the reference category. The main findings, which showed an odds ratio of around two, were as follows. Teachers showed a significantly higher adherence to calcium intake recommendations than nurses (OR, 2.54; 95% CI, 2.02–3.14; *p* < 0.001); agriculture workers showed significantly a higher adherence to calcium intake recommendations compared with forestry and fishery workers (OR, 2.15; 95% CI, 1.46–3.15; *p* < 0.001); agriculture workers also showed a significantly higher adherence to recommendations for vitamin C intake compared with forestry and fishery workers (OR 1.90; 95% CI 1.31–2.74; *p* = 0.001).

**Figure 4:**
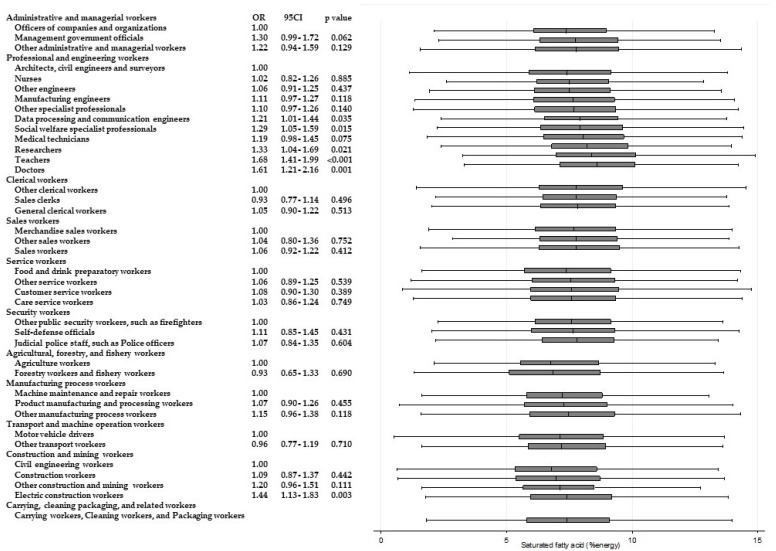
Daily saturated fatty acid intake according to occupational groups. OR: Odds ratio of daily energy intake from saturated fatty acid equal to or more than 7% energy.

**Figure 5:**
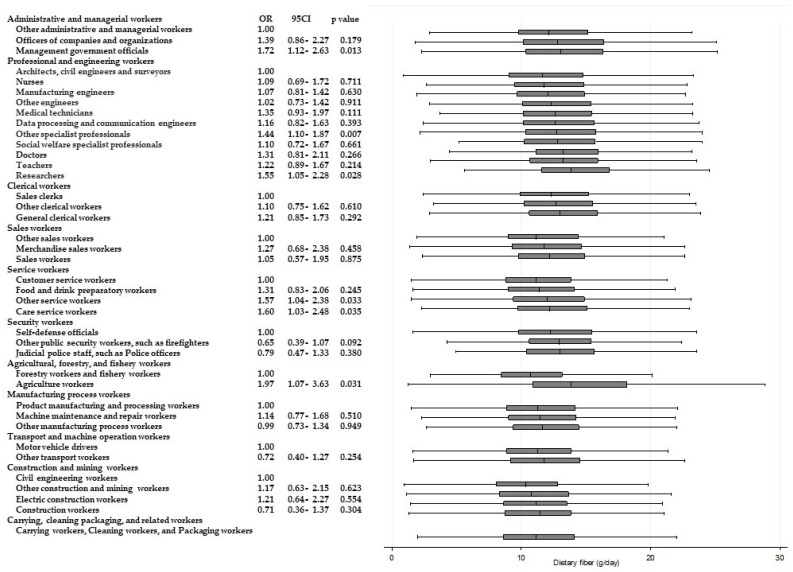
Daily dietary fiber intake according to occupational groups. OR: Odds ratio of daily dietary fiber intake equal to or more than 20 g.

**Figure 6:**
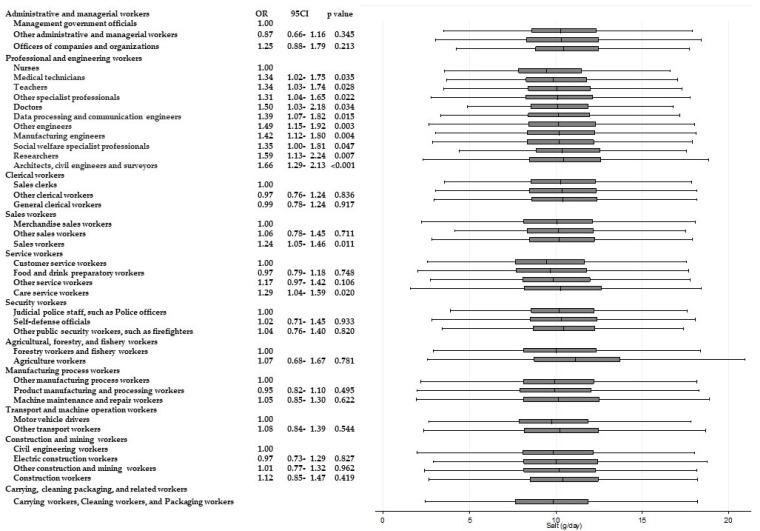
Daily salt intake according to occupational groups. OR: Odds ratio of daily salt intake equal to or more than 8 g.

**Figure 7:**
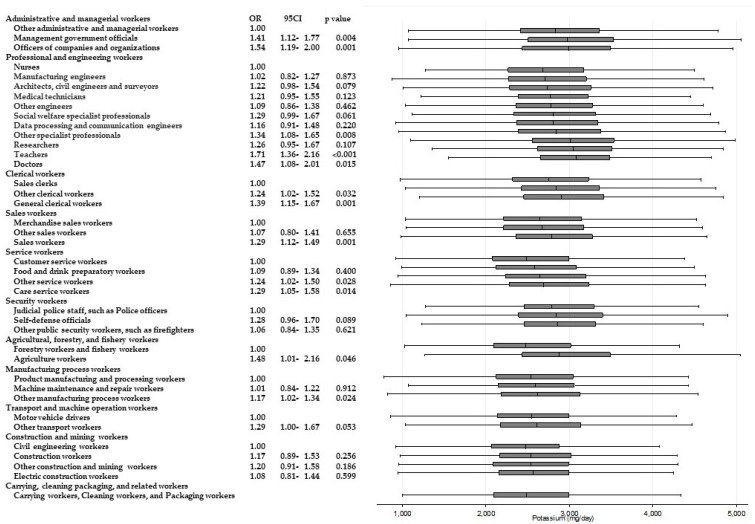
Daily potassium intake according to occupational groups. OR: Odds ratio of daily potassium intake equal to or more than 3000 mg.

**Figure 8:**
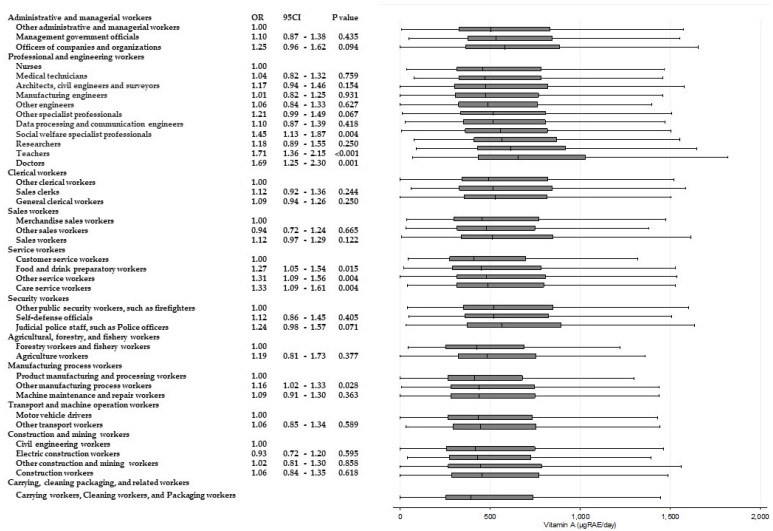
Daily vitamin A intake according to occupational groups. OR: Odds ratio of daily vitamin A intake equal to or more than 600 mg.

**Figure 9:**
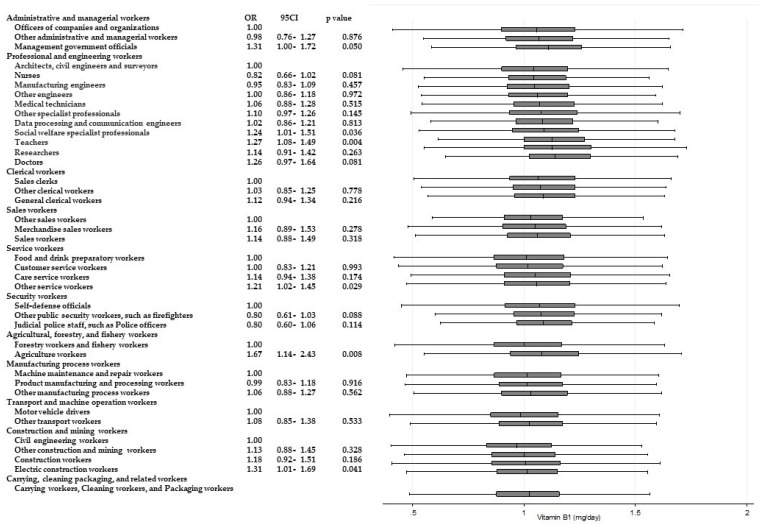
Daily vitamin B_1_ intake according to occupational groups. OR: Odds ratio of daily vitamin B_1_ intake equal to or more than 1.1 mg.

Significantly higher odds of intakes of other nutrients are also shown in Figure 4, Figure 5, Figure 6, Figure 7, Figure 8 and Figure 9. Teachers showed significantly higher odds of adherence to the recommended intake of potassium (OR 1.71; 95% CI 1.36–2.16; *p* < 0.001) and vitamin A (OR 1.71; 95% CI 1.36–2.15; *p* < 0.001) compared with nurses, and significantly higher odds of higher saturated fatty acid intake than recommended (lower adherence to recommendation) compared with architects, civil engineers, and surveyors (OR 1.68; 95% CI 1.41–1.99; *p* < 0.001). Architects, civil engineers, and surveyors in turn showed higher odds of higher salt intake than recommended (lower adherence to recommendation) compared with nurses (OR 1.66; 95% CI 1.29–2.13; *p* < 0.001).

## 4. Discussion

The present study found significant differences in dietary intake between smaller occupational groups (i.e., Minor or Unit Groups) within the larger occupational categories (i.e., Major Groups). Significant differences among all of the nutrients that were evaluated in this study were observed among “professional and engineering workers”. Similarly, significant differences were observed in all of the micronutrients that were evaluated among “service workers” and “agricultural, forestry, and fishery workers”, as well as “professional and engineering workers”. Regarding intake of calcium and vitamins A, B1, and C, the median values among some occupational groups were under the recommended levels, whereas among others, the median values were above recommended levels. For nutrient intake, higher odds ratios of adherence to recommendations, particularly for calcium and vitamin C, were observed among “professional and engineering workers” and “agricultural, forestry, fishery workers”. Notably, within the “professional and engineering workers” category, teachers had significantly higher odds compared with nurses. Similarly, in “agricultural, forestry, fishery workers”, agriculture workers had significantly higher odds compared with forestry and fishery workers.

In interpreting these findings, it is necessary to focus on what factors contribute to workers’ diets among each small occupational group. Educational level is known to be associated with dietary intake [15,16]; however, after adjustment for potential confounding factors including educational level, some associations between occupation and dietary intakes were still significant, suggesting that the dietary differences between occupational groups cannot be explained by only educational level. For example, nurses tended to show a lower adherence to recommendations on several nutrients than teachers after adjusting for potential confounding factors, including education. For reference, most nurses in this study graduated from a professional school, while teachers graduated from a university (data not shown); on the other hand, nurses are health professionals who should have higher than average knowledge of health behaviors. To interpret poor dietary intake among nurses, it may thus be necessary to focus on organizational factors that may impact diet regardless of their knowledge level.

Such organizational factors could include the nature of the work itself and/or the workplace environment. First, work-related factors may affect dietary behaviors and dietary intakes. For example, work hours [26], shift work [27,28,29], and work control [13] have all been reported to be associated with diet across various occupations. Among nurses, recent research has shown an association between shift schedules and eating behaviors [28,30]. In their review, Nicholls et al. also identified that organizational factors, including long working hours and shift work, seem to be barriers to a healthy diet for nurses [31]. Lower nutrient intake among nurses in the current study may be also explained by such work-related factors. Higher salt intakes among architects, civil engineers, and surveyors may also be explained by the work itself and/or the workplace environment. Among “professional and engineering workers”, architects, civil engineers, and surveyors may be relatively close to workers who are engaged in physically demanding jobs, such as construction workers; in fact, construction workers also showed higher salt intakes. Surveyors in particular could often be engaged in outdoor work while on the job, and architectural and civil engineers are often in contact with individuals working in construction; although they have different roles and responsibilities, they could often work in the same workplace.

Another explanation might be food availability and accessibility within these and other occupations. Some previous studies have found associations between food facilities in the workplace such as cafeterias [32,33,34] and vending machines [33] with diet food. Roos et al. suggest that staff canteens could contribute to healthy eating [34]. In contrast, Kjollesdal et al. found that frequent eating in staff canteens is associated with unhealthy eating patterns [35]. Accordingly, food environments and facilities in the workplace, such as cafeterias, shops, and vending machines, could contribute to workers’ healthy diet, but only if healthier food options are available. In Japan, the provision of school lunch with menus designed by nutritionists has been researched. Asakura et al. reported the important contribution of school lunch to developing children’s dietary habits [36]. The provision of school lunches may be a contributing factor to the higher nutrient intake among teachers as found in the current study as well. Similarly, higher nutrient intake among agricultural workers could also be explained by their food environment. Umezawa et al. suggest that growing one’s own vegetables contributes to a higher vegetable intake [37]. Additionally, such a food environment seems to be important in developing nutritional education. Sato argues that agricultural experience contributes to food choices [38]. It is worth noting that nutritional education can be conducted not only through textbook learning but also through experiential learning. Even if people are not engaged in agricultural work, they can still learn about the importance of healthy eating through workplace food facilities that provide healthy foods, and develop an awareness surrounding their food choices.

Considering the variations in factors related to work and workplace across occupations, it may be appropriate in future research to classify occupation using more specific techniques, such as Minor or Unit Groups, as reported here. In previous studies that have investigated occupational status as one of the socioeconomic factors, occupations have usually used only large and general categories, such as “professional” [11], “manual” [13], or “service workers” [14]. However, from an occupational health perspective, we should pay more attention to the occupational context, such as the type of occupation, work-related factors, and environmental factors in the workplace. Importantly, the results of the present study reveal that there are differences among occupations within the large occupational categories as they are typically used, especially within “professional and engineering workers” and “agricultural, forestry, and fishery workers”. Given the variations in occupational characteristics within the broad occupational categories, it is unlikely that all of the workers in each category should be considered in the same way. Therefore, detailed occupational classification may be useful in conducting dietary research, especially for “professional and engineering workers” and “agricultural, forestry, and fishery workers”.

In summary, the present study found clear dietary differences among large occupational groups when classified into smaller, more specific groups. Considering the background and contextual factors related to different occupations and dietary choices, it may be helpful to consider these in the development of guidance for health and diet, as appropriate for each group. Further studies are required to clarify which factors are most important in influencing workers’ diet according to their occupation.

## 5. Limitations

Several limitations of the current study should be acknowledged. First, this study did not consider dietary differences by gender, because only men were recruited as participants. Given the increasing number of working women, this research should be replicated for women as well. Furthermore, it may be difficult to apply the results of the present study to all working men, because the study subjects were limited to men whose partner was a pregnant woman. Second, the analyses were conducted based only on data from self-reported questionnaires. It is possible that over-reporting or under-reporting could occur, particularly for the Food Frequency Questionnaire, partly because social desirability bias may affect the participants’ responses. Third, due to issues associated with self-selection bias, it is possible that the participants of this study may have greater interest in health and diet compared with the general population; additionally, they may have been able to answer questions from a more informed perspective. Fourth, it is not possible to clarify the causal pathways between occupations and dietary intakes because of the cross-sectional design.

## 6. Conclusions

The current study identified that when using detailed occupational classification, significant differences in dietary intake can be detected among working men compared with using only large occupational categories. This more precise classification of occupational status allows for the detection of differences in dietary intake and may be helpful for future research, as well as informing the development of support promoting a healthy diet for workers.

## Figures and Tables

**Table 1:** Characteristics of the participants: occupational classifications (N = 38,656).

Occupation	N	%
Administrative and managerial workers		
Management government officials	603	1.56
Officers of companies and organizations	387	1.00
Other administrative and managerial workers	687	1.78
Professional and engineering workers		
Researchers ^a^	489	1.27
Manufacturing engineers	2332	6.03
Architects, civil engineers, and surveyors	1608	4.16
Data processing and communication engineers	897	2.32
Other engineers	1115	2.88
Doctors ^b^	389	1.01
Nurses	488	1.26
Medical technicians ^c^	718	1.86
Social welfare specialist professionals	543	1.40
Teachers ^d^	1238	3.20
Other specialist professionals	2490	6.44
Clerical workers		
General clerical workers	1923	4.97
Sales clerks	660	1.71
Other clerical workers	1233	3.19
Sales workers		
Merchandise sales workers	1379	3.57
Sales workers	2654	6.87
Other sales workers	274	0.71
Service workers		
Care service workers	943	2.44
Food and drink preparatory workers	973	2.52
Customer service workers	993	2.57
Other service workers	1310	3.39
Security workers		
Self-defense officials	461	1.19
Judicial police staff, such as police officers	616	1.59
Other public security workers, such as firefighters	648	1.68
Agricultural, forestry, and fishery workers		
Agriculture workers	401	1.04
Forestry workers and fishery workers	255	0.66
Manufacturing process workers		
Product manufacturing and processing workers	2897	7.49
Machine maintenance and repair workers	722	1.87
Other manufacturing process workers	1591	4.12
Transport and machine operation workers		
Motor vehicle drivers	1090	2.82
Other transport workers	536	1.39
Construction and mining workers		
Construction workers	754	1.95
Electric construction workers	585	1.51
Civil engineering workers	539	1.39
Other construction and mining workers	700	1.81
Carrying, cleaning packaging, and related workers		
Carrying workers, cleaning workers, and packaging workers	535	1.38

^a^ Researchers: natural science researchers, humanities, social science, and other researchers; ^b^ Doctors: doctors except for dental surgeons, veterinary surgeons, and pharmacists; ^c^ Medical technicians: diagnostic radiographers, clinical engineers, clinical laboratory technicians, physiotherapists, occupational therapists, certified orthoptists, speech therapists, dental hygienists, and dental technicians; ^d^ Teachers: kindergarten teachers, elementary school teachers, junior high school teachers, senior high school teachers, secondary educational school teachers, special needs education school teachers, vocational school teachers, university professors, and other teachers.

**Table 2:** Characteristics of the participants: socioeconomic factors and compliance with dietary recommendations (N = 38,656).

	N	%
Educational level		
Junior high school	1865	4.8
High school	13,632	35.0
Higher professional school	805	2.0
Professional school	7539	20.0
Junior college	837	2.0
University	11,935	31.0
Graduate school	2043	5.3
Household income		
<2 million yen	1541	4.0
2.0–3.9 million yen	12,908	33.4
4.0–5.9 million yen	13,280	34.4
6.0–7.9 million yen	6522	16.9
8.0–9.9 million yen	2735	7.1
10–11.9 million yen	988	2.6
12–14.9 million yen	366	1.0
15–19.9 million yen	215	0.6
≥20 million yen	101	0.3
Adherence to recommendations		
Protein 13–20% energy/day ^a^	14,705	38.0
Fatty acid 20–30% energy/day ^a^	21,130	55.0
Carbohydrate 50–65% energy/day ^a^	23,764	61.0
Saturated fatty acid ≤7 g/day ^a^	15,451	40.0
Dietary fiber ≥20 g/day ^a^	2349	6.1
Salt <8 g/day ^a^	8389	21.7
Potassium ≥3000 mg/day ^a^	13,639	35.3
Calcium ≥550 mg/day ^b^	16,711	43.2
Vitamin A ≥600 mg/day ^b^	14,744	38.1
Vitamin C ≥85 mg/day ^b^	16,905	44.0
Vitamin B1 ≥1.1 mg/day ^b^	16,110	41.7

^a^ Tentative dietary goals for preventing lifestyle-related diseases (DG); ^b^ Lowest value among estimated average requirements (EAR) for men aged 18–69.

**Table 3:** Differences in dietary intakes according to occupational groups: the results from analysis of variance (ANOVA) or *t*-test.

Energy and Nutrients	Energy	Protein	Fatty Acid	Carbohydrate	Saturated Fatty Acid	Dietary Fiber	Salt	Potassium	Calcium	Vitamin A	Vitamin C	Vitamin B_1_
Administrative and managerial workers ^a^	*	NS	NS	*	NS	*	NS	*	NS	NS	*	**
Professional and engineering workers ^a^	**	**	**	**	**	**	**	**	**	**	**	**
Clerical workers ^a^	NS	NS	NS	NS	NS	*	NS	**	*	NS	**	NS
Sales workers ^a^	*	*	NS	**	NS	*	NS	**	**	**	**	NS
Service workers ^a^	NS	*	NS	**	NS	**	**	**	**	**	**	**
Security workers ^a^	*	*	NS	*	NS	NS	NS	NS	NS	NS	*	NS
Agricultural, forestry, and fishery workers ^b^	NS	NS	NS	NS	NS	**	*	**	**	**	**	**
Manufacturing process workers ^a^	*	*	*	*	*	*	NS	**	NS	*	NS	*
Transport and machine operation workers ^b^	NS	**	*	NS	NS	NS	*	*	NS	NS	*	*
Construction and mining workers ^a^	NS	*	**	NS	**	*	NS	NS	*	NS	*	NS
Carrying, cleaning packaging, and related workers	-	-	-	-	-	-	-	-	-	-	-	-

* *p* < 0.05; ** *p* < 0.001; NS, not significant; ^a^ ANOVA was performed; ^b^ *t*-test was performed.

## References

[1] Estruch R., Ros E., Salas-Salvado J., Covas M.I., Corella D., Aros F., Gomez-Gracia E., Ruiz-Gutierrez V., Fiol M., Lapetra J. (2013). Primary prevention of cardiovascular disease with a Mediterranean diet. N. Engl. J. Med..

[2] Du H., Li L., Bennett D., Guo Y., Key T.J., Bian Z., Sherliker P., Gao H., Chen Y., Yang L. (2016). Fresh Fruit Consumption and Major Cardiovascular Disease in China. N. Engl. J. Med..

[3] Kwok C.S., Boekholdt S.M., Lentjes M.A., Loke Y.K., Luben R.N., Yeong J.K., Wareham N.J., Myint P.K., Khaw K.T. (2015). Habitual chocolate consumption and risk of cardiovascular disease among healthy men and women. Heart.

[4] Aune D., Keum N., Giovannucci E., Fadnes L.T., Boffetta P., Greenwood D.C., Tonstad S., Vatten L.J., Riboli E., Norat T. (2016). Whole grain consumption and risk of cardiovascular disease, cancer, and all cause and cause specific mortality: Systematic review and dose-response meta-analysis of prospective studies. BMJ.

[5] Mozaffarian D. (2016). Dietary and Policy Priorities for Cardiovascular Disease, Diabetes, and Obesity: A Comprehensive Review. Circulation.

[6] Geaney F., Kelly C., Greiner B.A., Harrington J.M., Perry I.J., Beirne P. (2013). The effectiveness of workplace dietary modification interventions: A systematic review. Prev. Med..

[7] Lin W., Hang C.M., Yang H.C., Hung M.H. (2011). 2005–2008 Nutrition and Health Survey in Taiwan: The nutrition knowledge, attitude and behavior of 19–64 year old adults. Asia Pac. J. Clin. Nutr..

[8] OECD Labour Force Participation Rate (Indicator). https://data.oecd.org/emp/labour-force-participation-rate.htm.

[9] Statistics Bureau, Ministry of Internal Affairs and Communications Employment Status Survey, 2012 Survey, Summary of the Results. http://www.stat.go.jp/english/data/shugyou/index.htm.

[10] Si Hassen W., Castetbon K., Cardon P., Enaux C., Nicolaou M., Lien N., Terragni L., Holdsworth M., Stronks K., Hercberg S. (2016). Socioeconomic Indicators Are Independently Associated with Nutrient Intake in French Adults: A DEDIPAC Study. Nutrients.

[11] Mishra G., Ball K., Patterson A., Brown W., Hodge A., Dobson A. (2005). Socio-demographic inequalities in the diets of mid-aged Australian women. Eur. J. Clin. Nutr..

[12] Kachan D., Lewis J.E., Davila E.P., Arheart K.L., LeBlanc W.G., Fleming L.E., Caban-Martinez A.J., Lee D.J. (2012). Nutrient intake and adherence to dietary recommendations among US workers. J. Occup. Environ. Med..

[13] Raberg Kjollesdal M.K., Holmboe-Ottesen G., Wandel M. (2010). Associations between food patterns, socioeconomic position and working situation among adult, working women and men in Oslo. Eur. J. Clin. Nutr..

[14] Fukuda Y., Nakamura K., Takano T. (2005). Accumulation of health risk behaviours is associated with lower socioeconomic status and women’s urban residence: A multilevel analysis in Japan. BMC Public Health.

[15] Mejean C., Si Hassen W., Lecossais C., Alles B., Peneau S., Hercberg S., Castetbon K. (2016). Socio-economic indicators are independently associated with intake of animal foods in French adults. Public Health Nutr..

[16] Boylan S., Lallukka T., Lahelma E., Pikhart H., Malyutina S., Pajak A., Kubinova R., Bragina O., Stepaniak U., Gillis-Januszewska A. (2011). Socio-economic circumstances and food habits in Eastern, Central and Western European populations. Public Health Nutr..

[17] Tsiga E., Panagopoulou E., Niakas D. (2015). Health promotion across occupational groups: One size does not fit all. Occup. Med..

[18] Communications, Ministry of Internal Affairs and Communications Japan Standard Occupational Classification (Rev. 5 December 2009) General Principles for the Japan Standard Occupational Classification. http://www.soumu.go.jp/english/dgpp_ss/seido/shokgyou/co09-2.htm.

[19] Communications, Ministry of Internal Affairs and Communications Japan Standard Occupational Classification (Rev. 5 December 2009) Structure and Explanatory Notes. http://www.soumu.go.jp/english/dgpp_ss/seido/shokgyou/co09-4.htm.

[20] Kawamoto T., Nitta H., Murata K., Toda E., Tsukamoto N., Hasegawa M., Yamagata Z., Kayama F., Kishi R., Ohya Y. (2014). Rationale and study design of the Japan environment and children’s study (JECS). BMC Public Health.

[21] Michikawa T., Nitta H., Nakayama S.F., Ono M., Yonemoto J., Tamura K., Suda E., Ito H., Takeuchi A., Kawamoto T. (2015). The Japan Environment and Children’s Study (JECS): A Preliminary Report on Selected Characteristics of Approximately 10,000 Pregnant Women Recruited during the First Year of the Study. J. Epidemiol..

[22] Michikawa T.N.H., Nakayama F.S., Yamazaki S., Isobe T., Tamura K., Suda E., Ono M., Yonemoto J., Iwai-Shimada M., Kobayashi Y. (2018). Baseline profile of participants in the Japan Environment and Children’s Study (JECS). J. Epidemiol..

[23] Kobayashi S., Asakura K., Suga H., Sasaki S. (2017). Living status and frequency of eating out-of-home foods in relation to nutritional adequacy in 4017 Japanese female dietetic students aged 18–20 years: A multicenter cross-sectional study. J. Epidemiol..

[24] Ministry of Health, Labour and Welfare Overview of Dietary Reference Intakes for Japanese. http://www.mhlw.go.jp/file/06-Seisakujouhou-10900000-Kenkoukyoku/Overview.pdf.

[25] Yokoyama Y., Takachi R., Ishihara J., Ishii Y., Sasazuki S., Sawada N., Shinozawa Y., Tanaka J., Kato E., Kitamura K. (2016). Validity of Short and Long Self-Administered Food Frequency Questionnaires in Ranking Dietary Intake in Middle-Aged and Elderly Japanese in the Japan Public Health Center-Based Prospective Study for the Next Generation (JPHC-NEXT) Protocol Area. J. Epidemiol..

[26] Escoto K.H., Laska M.N., Larson N., Neumark-Sztainer D., Hannan P.J. (2012). Work hours and perceived time barriers to healthful eating among young adults. Am. J. Health Behav..

[27] Hemio K., Puttonen S., Viitasalo K., Harma M., Peltonen M., Lindstrom J. (2015). Food and nutrient intake among workers with different shift systems. Occup. Environ. Med..

[28] Han K., Choi-Kwon S., Kim K.S. (2016). Poor dietary behaviors among hospital nurses in Seoul, South Korea. Appl. Nurs. Res..

[29] Ramin C., Devore E.E., Wang W., Pierre-Paul J., Wegrzyn L.R., Schernhammer E.S. (2015). Night shift work at specific age ranges and chronic disease risk factors. Occup. Environ. Med..

[30] Almajwal A.M. (2016). Stress, shift duty, and eating behavior among nurses in Central Saudi Arabia. Saudi Med. J..

[31] Nicholls R., Perry L., Duffield C., Gallagher R., Pierce H. (2017). Barriers and facilitators to healthy eating for nurses in the workplace: An integrative review. J. Adv. Nurs..

[32] Raulio S., Roos E., Prattala R. (2010). School and workplace meals promote healthy food habits. Public Health Nutr..

[33] Almeida F.A., Wall S.S., You W., Harden S.M., Hill J.L., Krippendorf B.E., Estabrooks P.A. (2014). The association between worksite physical environment and employee nutrition, and physical activity behavior and weight status. J. Occup. Environ. Med..

[34] Roos E., Sarlio-Lahteenkorva S., Lallukka T. (2004). Having lunch at a staff canteen is associated with recommended food habits. Public Health Nutr..

[35] Kjollesdal M.R., Holmboe-Ottesen G., Wandel M. (2011). Frequent use of staff canteens is associated with unhealthy dietary habits and obesity in a Norwegian adult population. Public Health Nutr..

[36] Asakura K., Sasaki S. (2017). School lunches in Japan: Their contribution to healthier nutrient intake among elementary-school and junior high-school children. Public Health Nutr..

[37] Umezawa A., Miwa T., Shibui E., Namikawa T., Tanaka N., Ishikawa M. (2012). Total Vegetable Intake and Homegrown Vegetable Intake in the Rural Area Residents of Hokkaido. Jpn. J. Nutr. Diet..

[38] Sato K. (2015). The influence an agricultural experience on eating habits in university student’s from an elementary school. J. Jpn. Mibyou Syst. Assoc..

